# Diffuse pulmonary small nodular and patchy infiltrates on chest X-ray with hemoptysis: TB or not TB?-a call for scale up of respiratory medicine services in African TB high burden countries: a case of idiopathic pulmonary hemosiderosis

**DOI:** 10.11604/pamj.2018.30.121.12691

**Published:** 2018-06-13

**Authors:** Kerclin Danielle de Klerk, Steffen Bau, Gunar Günther

**Affiliations:** 1 Katutura State Hospital, Windhoek, Namibia; 2 Lady Pohamba Private Hospital, Windhoek, Namibia; 3 Katutura State Hospital, UNAM School of Medicine, Windhoek, Namibia; 4 Research Center Borstel, Borstel, Germany

**Keywords:** Pulmonary tuberculosis, idiopathic pulmonary haemo-siderosis, respiratory services

## Abstract

Tuberculosis is still one of the most common respiratory diseases in Africa and worldwide and miliary tuberculosis is a regular manifestation of it. Idiopathic pulmonary hemosiderosis is a rare disease entity, presenting in children as well as adults. It is characterized by the triad of recurrent episodes of alveolar hemorrhage, presenting as hemoptysis, iron deficiency anemia and bilateral pulmonary infiltrates seen on chest X-ray. These symptoms and signs can easily be confused for other diseases i.e. miliary tuberculosis, delaying appropriate management. The etiology of idiopathic pulmonary hemosiderosis remains unclear. Diagnosis is established by lung biopsy, revealing hemosiderin laden macrophages in the alveoli. Treatment during an acute episode includes corticosteroids and/or immunosuppressive therapy, as well as supportive measures. Long-term follow-up is essential.

## Introduction

The case describes the presumed diagnosis of miliary tuberculosis (TB) in an 11-year-old, immunocompetent boy based on chest X-ray changes and clinical symptoms. The high incidence of TB in Namibia and hence high pretest probability made the diagnosis likely, despite consistently negative cultures. Failure of clinical response should have made the treating doctors question the diagnosis much earlier. Only upon referral to a specialist service with scrutinizing of the history followed by diagnostic opportunities of high resolution computed tomography (HR-CT), bronchoscopy and trans-bronchial biopsy made the alternative diagnosis of idiopathic pulmonary hemosiderosis (IPH) possible.

## Patient and observation

**Figure 1:**
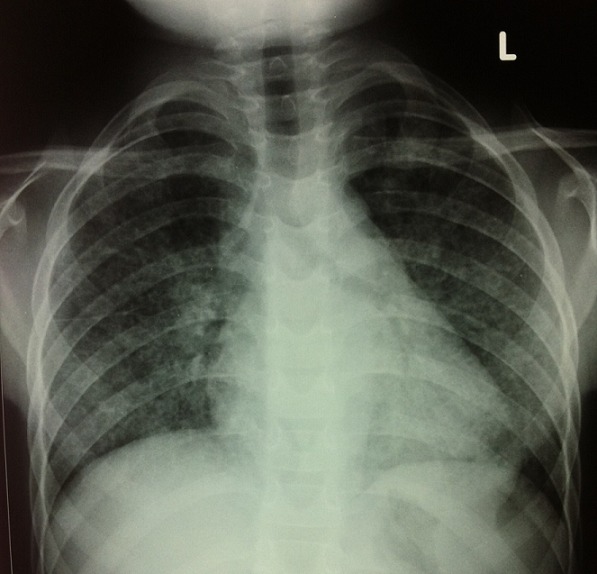
Postero-anterior chest X-rays showing bilateral diffuse nodular infiltrates, similar to miliary TB

**Figure 2:**
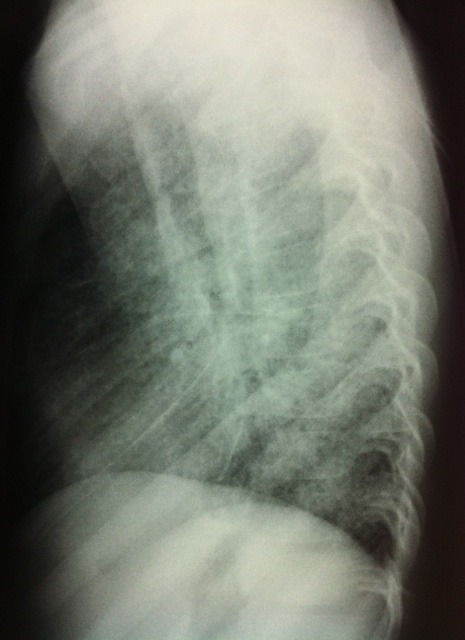
Lateral chest X-rays showing bilateral diffuse nodular infiltrates, similar to miliary TB

**Figure 3:**
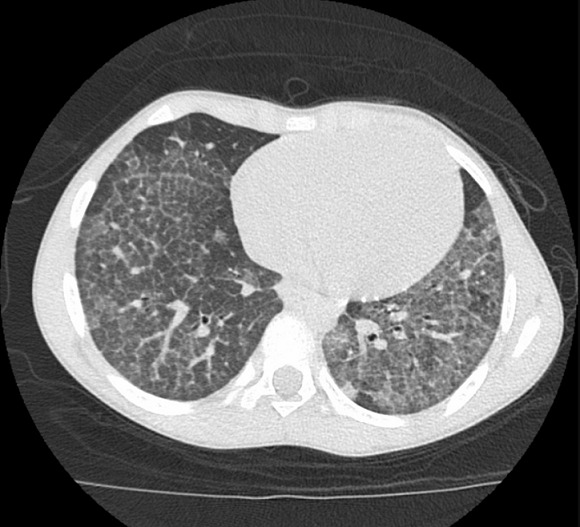
HR-CT scan showing diffuse ground glass opacities indicative of alveolar haemorrhage and thickened interlobar septae

**Figure 4:**
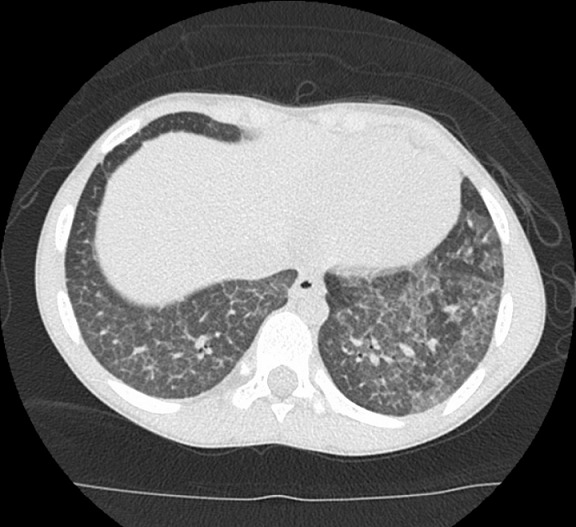
HR-CT scan showing diffuse ground glass opacities indicative of alveolar haemorrhage and thickened interlobar septae, indicating extent of disease

**Figure 5:**
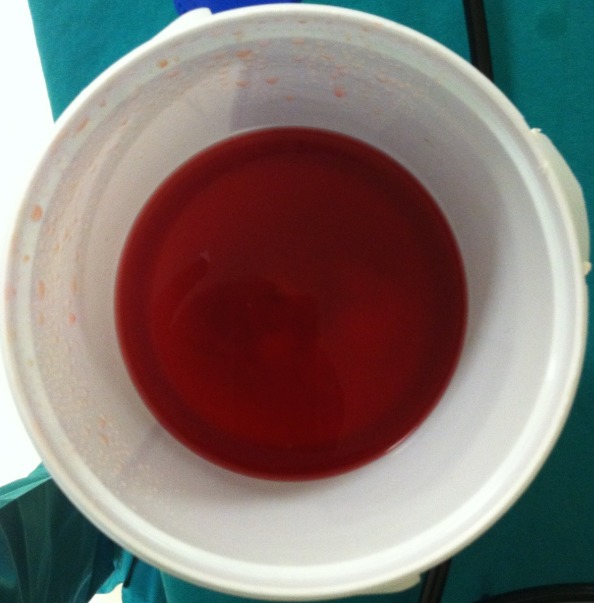
Bilateral alveolar lavage showing diffuse macroscopic haemorrhage

**Figure 6:**
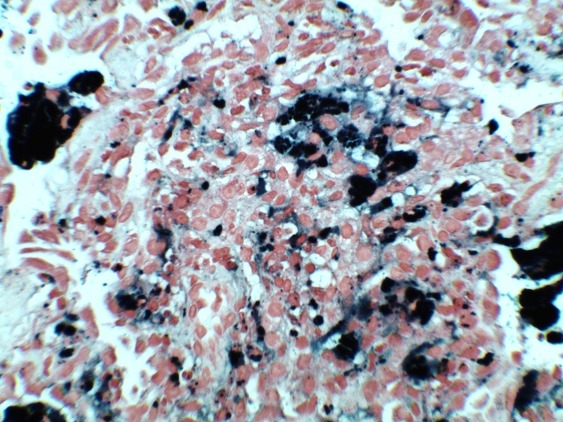
Histology of trans-bronchial biopsy demonstrating iron laden macrophages in silver stain

**Figure 7:**
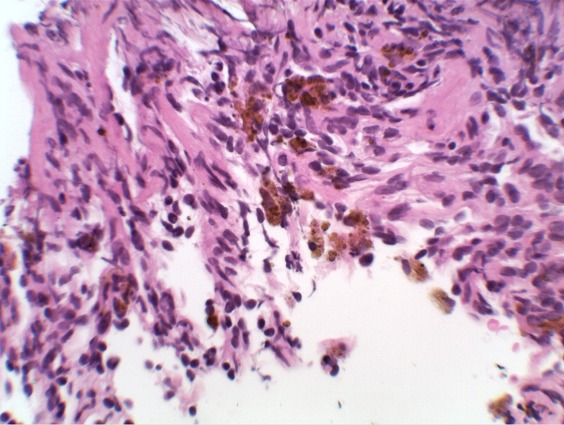
Histology of trans-bronchial biopsy demonstrating iron laden macrophages H&E stain

An 11-year-old boy who presented with a four-year history of hypoxemia, ongoing hemoptysis and easy fatigability. His chest X-ray showed diffuse nodular infiltrates, resembling a pattern interpretable as miliary tuberculosis (Figure 1, Figure 2). The patient was repeatedly treated for pulmonary TB for a total period of three years, without treatment response. TB cultures and smears remained negative. Upon referral to a recently established specialist respiratory service, a HR-CT scan showed diffuse ground glass infiltrates (Figure 3, Figure 4). Consecutive bilateral alveolar lavage showed diffuse pulmonary hemorrhage (Figure 5), while histology of trans-bronchial biopsy demonstrated iron loaded pulmonary macrophages (Figure 6, Figure 7). The findings confirm the diagnosis of IPH. During his course of disease, the boy was admitted and investigated several times at his local hospital. This included treatment in an ICU in 2008, where he was diagnosed with severe pneumonia, pulmonary edema and respiratory failure. History reported an ongoing non-productive cough for one week, shortness of breath, hemoptysis and generalized weakness. On presentation severe respiratory distress, hypoxemia and pyrexia were reported. Treatment included a course of broad spectrum antibiotics, furosemide and steroids. The patient was started on TB treatment after discharge (he had a positive TB contact) despite that TB smear, microscopy and repeated cultures remained negative. He had received a blood transfusion after laboratory investigations revealed a hemoglobin (Hb) level of 6.60 g/dl, with a mean cell volume (MCV) 65fl and mean cell hemoglobin (MCH) 17.4 pg, in keeping with iron deficiency anemia. Further investigations showed normal liver function, renal function, serum electrolytes and clotting profile. HIV serology, auto-immune screen (ANA, anti-DS DNA, RF) and TB Mantoux test were negative. After discharge from ICU he continued TB treatment, but without substantial improvement of symptoms and more admissions to the local hospital. After three years he was referred for a second opinion to a pediatrician and onwards to a specialist respiratory service, which allowed the diagnosis of IPH. At referral the patient had an acute episode with deterioration of oxygen saturation from above 85% to the 60-75%. The chest X-ray demonstrated a miliary picture (Figure 1). After establishing the diagnosis, hypoxia and X-ray changes resolved after a week of high dose steroids (2mg/kg prednisolone) and he was discharged home without home oxygen. Outpatient treatment included milk-free diet, tapered doses of steroids for two months, followed by inhaled budesonide 400 mcg BD and hydroxychloroquine which was discontinued after his eyesight deteriorated at six months. One year after diagnosis he remains in remission on inhaled corticosteroids and milk-free diet.

## Discussion

Miliary TB is characterized by the hematogenous spread of TB and typical chest X-ray findings of small nodular infiltrates. It seems only evident in less than 2% of TB cases and can on its radiological and clinical appearance be confused with numerous other pulmonary conditions [1]. In children, IPH occurs in equal frequency in the two genders; in adulthood the disease favours males [2]. Studies suggest that genetics play a role in the development of IPH, with environmental factors precipitating the disease in predisposed individuals. Such factors include exposure to insecticides, moulds and fungi, with the latter having a more prominent role. *Stachybotrys atra*, a mould, was isolated in most cases. The spores produce toxins called thrichothecenes, which are potent protein synthesis inhibitors. They impede angiogenesis and make capillaries fragile, leaving patients at risk for stress hemorrhage [3]. Other suspected aetiologies include allergic and auto-immune mediated mechanisms, supported by the frequent findings of associated cow milk protein allergy (Heiner syndrome) or of other autoimmune diseases like celiac disease, glomerulonephritis and rheumatoid arthritis. The exact etiology remains however unknown [4]. *Clinically*, childhood IPH presents before 10 years. Typical symptoms include dyspnea and recurrent cough, which is initially irritative and then progresses to hemoptysis, with episodes of overt bleeding from the lower respiratory tract. Children fail to thrive and may develop moderate to severe anemia, with features of iron deficiency. Blood stained sputum can occasionally be swallowed and can test positive for occult blood in the feces. Hepato- and splenomegaly and generalized lymphadenopathy is an occasional finding. In adults, symptoms include exertional dyspnea and fatigue due to intrapulmonary bleeding, and iron deficiency anemia. Hemoptysis is very common but may range from occasional blood-stained sputum to frank bleeding. Long standing disease can lead to the development of pulmonary fibrosis [2]. On *iron studies*, IPH shows low serum iron with low iron saturation; on blood film it shows microcytosis and hypochromia. Hypoferritinemia is commonly used as a diagnostic marker for iron deficiency. However, being an acute phase reactant, it can be abnormally increased in acute infection and liver disease [5]. Plasma ferritin levels can be normal or elevated because of alveolar synthesis and release into circulation. This makes the diagnosis of iron deficiency anemia difficult. It is therefore recommended that serum iron and transferrin saturation be used to evaluate iron deficiency anemia [6]. There is no pathognomonic finding for IPH regarding imaging *studies*. During acute phase exacerbations, chest X-rays typically show diffuse alveolar type infiltrates, predominantly in the lower lung fields. During remission, the alveolar infiltrates get absorbed and interstitial reticular and micronodular patterns of opacities ensue [6]. This corresponds to the ground glass attenuation seen on HR-CT scan. Lung function tests shows impaired function with a restrictive pattern. Respiratory insufficiency can occur, manifesting at rest or on exertion [2].

The *diagnostic approach* encompasses two stages: one- proof of diffuse intrapulmonary bleeding, i.e the patient's clinical picture; cough, hemoptysis, dyspnea, secondary iron deficiency anemia, pallor, failure to thrive, multiple alveolar type opacities, erythrocytes and siderophages in sputum and bilateral alveolar lavage (BAL). Postero-anterior and lateral chest X-rays is standard procedure, followed by HR-CT scan in order for better characterization and localization of the area of alveolar bleeding and assessing the degree of interstitial fibrosis. A full anemic work-up, which includes gastrointestinal and genitourinary work-up for occult bleeding and iron studies should be included [2]. Secondly, exclude any other conditions that are associated with diffuse alveolar hemorrhage (DAH), e.g systemic and autoimmune diseases. A bronchial alveolar lavage, demonstrating DAH, and trans-bronchial biopsy can confirm the diagnosis. Recent or active diffuse alveolar bleeding can be seen by the presence of intact or minimally fragmented erythrocytes in the alveoli and distal airways, while the presence of multiple hemosiderin-laden alveolar macrophages called siderophages is an expression of subacute/chronic or recurrent intrapulmonary bleeding [2,7]. The current *management of IPH* is based on small observational studies. The use of systemic corticosteroids has been under scrutiny in therapeutic trials and have shown to be more beneficial in the acute phase [2,8]. The recommended starting dose in the initial phase is high (e.g prednisone 2-5mg/kg/d, or equivalent). This is continued until the alveolar infiltrates resolve, and then slowly tapered providing that symptoms do not recur. It has been shown that most patients have responded favourably with chronic oral corticosteroid use, with decreased incidence of acute exacerbations and a decline in fibrogenesis. However, the side effects and recurrence of IPH during the tapering phase is problematic. Inhaled corticosteroids have also been tried, but there is lack of sufficient evidence for their efficacy. [9] Other immunosuppressive therapies, such as azathioprine, methotrexate, cyclophosphamide and hydroxychloroquine have been used, with variable results. Among these agents, azathioprine in combination with oral corticosteroids has been shown to be the best therapeutic regimen, especially with regards to prevention of acute exacerbations [10]. An improvement in morbidity and mortality is possibly due to the long-term use of such therapies [7]. Long term *follow-up* should encompass the number and severity of hemorrhage episodes, progression of interstitial lung fibrosis and clinical response to treatment. This can be measured against an improvement in symptoms, anemia, radiological infiltrates and return to baseline of pulmonary function tests [10]. The *prognosis* of IPH is variable, and due to the lack of large and comparable patient studies and poor follow-up, it remains unclear. A survival time of five years in 86% of patients have been reported when treated with long term immunosuppressive therapy. It has shown that aggressive treatment in IPH will have a longer survival and better prognosis [10].

## Conclusion

In conclusion, pulmonary TB, including atypical presentations on chest X-ray, is common in TB high incidence countries but alternative diagnoses must always be considered. In our case based on the symptoms and signs, empiric TB treatment, even while culture negative, in the absence of other diagnostic means was probably initially appropriate. But the non-response to therapeutic interventions on a presumed diagnosis should lead to early referral to specialist care. Unfortunately, i.e specialist respiratory services are rarely established in sub-Saharan Africa despite the high burden of respiratory diseases.

## Competing interests

The author declare no competing interest.
